# The miRNA Transcriptome Directly Reflects the Physiological and Biochemical Differences between Red, White, and Intermediate Muscle Fiber Types

**DOI:** 10.3390/ijms16059635

**Published:** 2015-04-29

**Authors:** Jideng Ma, Hongmei Wang, Rui Liu, Long Jin, Qianzi Tang, Xun Wang, Anan Jiang, Yaodong Hu, Zongwen Li, Li Zhu, Ruiqiang Li, Mingzhou Li, Xuewei Li

**Affiliations:** 1 Institute of Animal Genetics & Breeding, College of Animal Science & Technology, Sichuan Agricultural University, Ya’an 625014, Sichuan, China; E-Mails: jideng_ma@sina.com (J.M.); hongmei_wang0826@163.com (H.W.); rui_liu777@163.com (R.L.); longjin8806@163.com (L.J.); wupie@163.com (Q.T.); xun_wang007@163.com (X.W.); lingdang317@163.com (A.J.); yaodong_hu@163.com (Y.H.); zhuli7508@163.com (L.Z.); 2 Farm Animal Genetic Resources Exploration and Innovation Key Laboratory of Sichuan Province, Sichuan Agricultural University, Ya’an 625014, Sichuan, China; 3 Novogene Bioinformatics Institute, Beijing 100083, China; E-Mails: lizongwen@novogene.cn (Z.L.); lirq@novogene.cn (R.L.)

**Keywords:** miRNA, fiber type, pig, myogenesis, energy metabolism

## Abstract

MicroRNAs (miRNAs) are small non-coding RNAs that can regulate their target genes at the post-transcriptional level. Skeletal muscle comprises different fiber types that can be broadly classified as red, intermediate, and white. Recently, a set of miRNAs was found expressed in a fiber type-specific manner in red and white fiber types. However, an in-depth analysis of the miRNA transcriptome differences between all three fiber types has not been undertaken. Herein, we collected 15 porcine skeletal muscles from different anatomical locations, which were then clearly divided into red, white, and intermediate fiber type based on the ratios of myosin heavy chain isoforms. We further illustrated that three muscles, which typically represented each muscle fiber type (*i.e.*, red: *peroneal longus* (PL), intermediate: *psoas major* muscle (PMM), white: *longissimus dorsi* muscle (LDM)), have distinct metabolic patterns of mitochondrial and glycolytic enzyme levels. Furthermore, we constructed small RNA libraries for PL, PMM, and LDM using a deep sequencing approach. Results showed that the differentially expressed miRNAs were mainly enriched in PL and played a vital role in myogenesis and energy metabolism. Overall, this comprehensive analysis will contribute to a better understanding of the miRNA regulatory mechanism that achieves the phenotypic diversity of skeletal muscles.

## 1. Introduction

Skeletal muscle is the major organ, by weight, in the body, accounting for approximately 40% of the body’s mass. It plays an important role in exercise and energy metabolism [1], and is a heterogeneous tissue comprising fibers that can be broadly classified as red (oxidative), intermediate (oxidative-glycolytic), and white (glycolytic) fiber types. Each fiber type is characterized by increased levels of different types of myosin heavy chain (MHC): red fibers, *Myh7* and *Myh2*; intermediate fibers, *Myh1*; and white fibers, *Myh4* [2,3,4]*.* Red fibers contain higher levels of mitochondria, capillaries, myoglobin, and lipids than white fibers. White fibers have higher levels of glycolytic enzymes than red fibers; for example, lactate dehydrogenase A (LDHA), which is one of the key metabolic enzymes for glycolysis in skeletal muscle [3]. Intermediate fibers have intermediate characteristics between red and white fibers, and display both oxidative and glycolytic capacities. The diversity of muscle fibers play important roles in metabolic health and disease. Whole-body insulin sensitivity and insulin-stimulated glucose transport are positively correlated with the proportion of oxidative fibers [5], while glycolytic muscle fibers show greater atrophy than the oxidative fibers in response to food deprivation [6].

miRNAs are small non-coding RNAs of ~22 nucleotides (nt) in length that regulate gene expression by specifically binding target mRNA and mediating mRNA degradation and/or translational inhibition. Emerging evidence has demonstrated that miRNAs play a critical role in skeletal muscle differentiation and metabolism [7,8]. Recently, several studies have found that, because of the metabolic needs of oxidative and glycolytic skeletal muscles, they shared most muscle-specific miRNAs that expressed at distinct levels [9,10]. Interestingly, miR-499 and miR-208b are positively associated with oxidative red fibers as they repress transcriptional repressors of slow-twitch contractile protein genes, such as *Sox6* [11].

Pigs (*sus scrofa*) have considerable agricultural significance in meat production. Skeletal muscle is a highly heterogeneous tissue, nonetheless previous studies of miRNA trancriptome differences only focused on two fiber types. To better understand and elucidate the major determinants for phenotypic properties of various muscle types at the miRNA level, we screened and selected three muscles that typically represented each muscle fiber type (*i.e.*, red, intermediate, and white) from 15 candidates, based on differences in their muscle fiber composition and metabolic capacity, and then investigated the differences in their miRNA transcriptomes using a deep sequencing approach. Illuminating the miRNA-based post-transcriptional regulatory mechanism in different fiber types will enrich our knowledge of the roles of miRNA in muscle biology, and help us to further understand the characteristics of distinct muscle fiber types.

## 2. Results and Discussion

### 2.1. The Characteristics of Skeletal Muscle Fiber Types

**Figure 1:**
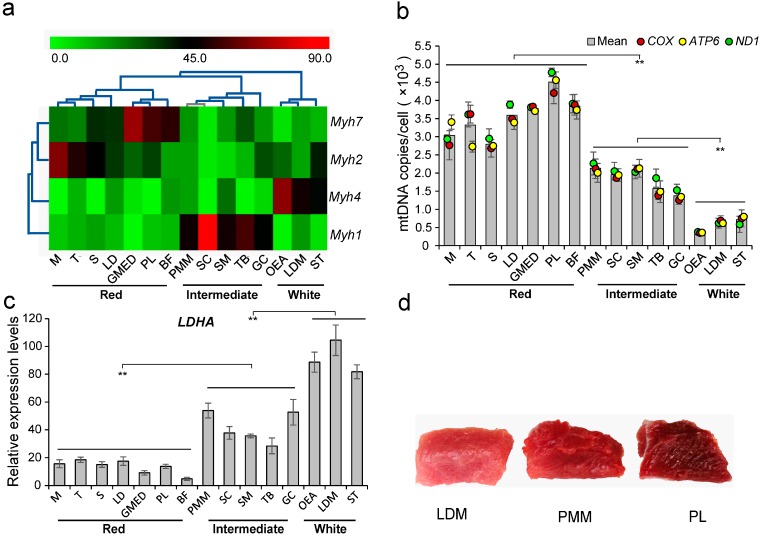
The characterization of various skeletal muscles. (**a**) Hierarchical clustering analysis for the ratios of myosin heavy chain isoforms in 15 muscle tissues based on Pearson correlation. M, *masseter*; T, *trapezius*; S, *soleus*; LD, *latissimus dorsi*; GMED, *gluteus medius*; PL, *peroneal longus*; BF, *biceps femoris*; PMM, *psoas major* muscle; SC, *semispinalis capitis*; SM, *semimembranosus*; TB, *triceps brachii*; GC, *gastrocnemius*; OEA, *obliquus externus abdominis*; LDM, *longissimus dorsi* muscle; ST, *semitendinosus*; (**b**) mtDNA copies per cell in 15 muscle tissues (*p* < 0.01); (**c**) The relative expression of lactose dehydrogenase A (*LDHA*) in 15 muscle tissues (*p* < 0.01); (**d**) The colors of LDM, PMM, and PL. Data are means ± SD. Statistical significance was calculated by one-way repeated-measures ANOVA (*n* = 3 per individual). ** *p* < 0.001.

To determine the muscles that typically represent each muscle fiber type, we collected 15 porcine skeletal muscles in different anatomical locations. qRT-PCR was performed to quantify the content of four MHC isoforms (*Myh1*, *Myh2*, *Myh4*, and *Myh7* genes) in 15 skeletal muscles. Although the mRNA sequences of these four genes showed high identity (>75%) with each other, Sanger sequencing for the PCR products of MHC isoforms confirmed the specificity and reliability of our qRT-PCR primers (data not shown). As shown in Figure 1a, hierarchical clustering analysis showed that distinct muscle types are divided into three clusters: red fiber (*Myh7* and *Myh2*), intermediate fiber (*Myh1*), and white fiber (*Myh4*) based on the ratios of MHC isoforms, which were consistent with previous classification of muscle fibers [12,13,14]. Meanwhile, mitochondrial contents and relative expression levels of *LDHA* were measured to distinguish the differences in metabolic capacity for distinct muscle fibers. Results showed that red fibers have the highest mtDNA copy number (Figure 1b), while white fibers have the highest *LDHA* expression levels (Figure 1c). Intermediate fibers exhibited intermediate levels for both measures.

Among these muscles, *peroneal longus* (PL) contained the highest number of copies of mtDNA per cell and lower *LDHA* expression (Figure 1b), suggesting its higher oxidative capacity compared to other skeletal muscles. In contrast, the *longissimus dorsi* muscle (LDM) exhibited the highest abundance of *LDHA* expression and relatively lower mtDNA copy number, suggesting it to be more proficient in anaerobic glycolytic metabolism (Figure 1c). Intriguingly, the *psoas* major muscle (PMM) that was regarded as a typical red muscle fiber type previously [4,15,16,17], was found to be an intermediate muscle fiber type (Figure 1a) in this study, with moderate mtDNA copy number and *LDHA* expression level. Moreover, the color of PMM was intermediate between red (PL) and white (LDM), which further confirmed the intermediate phenotype of PMM (Figure 1d). Therefore, PL, PMM, and LDM were selected as the most representative muscles for red, intermediate, and white fiber types respectively, in the subsequent analyses.

### 2.2. Summary of Deep Sequencing Data

To further identify the miRNA transcriptome differences between the three fiber types (*i.e.*, PL, PMM, and LDM), a deep sequencing approach was applied [18]. As a result, we obtained 9.17 million (M), 18.46 M, and 16.62 M raw reads for PL, PMM, and LDM, respectively. More than 98.58% (98.83% ± 0.26%, *n* = 3) of the raw reads in each library passed the quality filters (see Experimental Section) and were considered “mappable reads”. Length distribution analysis showed that the majority of reads ranged from 21 to 23 nt in length, and the 22 nt small RNA was the most abundant (61.50%), followed by 21 nt (13.37%) and 23 nt (18.33%) (Figure S1a). These results indicate the reliability of our small RNA sequencing, thus the mappable reads were selected as reliable miRNA candidates for subsequent analysis.

The vast majority (81.14%) of the mappable sequences were mapped to known precursor miRNAs (pre-miRNA) (miRBase19.0) (Table S1). The identified precursor and mature miRNAs were then divided into three groups using the following alignment criteria: (1) Known porcine miRNAs: 424 miRNAs mapped to 342 porcine known pre-miRNAs (Table S2.1); (2) Conserved miRNAs: 152 miRNAs mapped to 135 other known mammalian pre-miRNAs and these pre-miRNAs then mapped to the pig genome (Table S2.2); (3) Candidate miRNAs: 397 miRNAs (longer than 18 nt and unmapped to any known mammalian pre-miRNAs) encompassing 329 candidate pre-miRNAs that were predicted RNA hairpins derived from the pig genome (Table S2.3). Notably, there are the distinct pre-miRNAs coding the identical mature miRNAs, which resulted in 973 miRNAs (*i.e.*, reference sequence) corresponding to 912 unique miRNA sequences. Known porcine miRNAs represented by three or more sequence reads (*n* = 365) were used for the following analyses to ensure the high reliability of the reported results (Table S3).

Notably, 79.35% (292 out of 365) of the known unique porcine miRNAs were expressed in all three libraries, while only 4, 26, and 6 of the unique miRNAs were specifically expressed in PL, PMM, and LDM, respectively, and the vast majority of these tissue-specific miRNAs were at low abundance (3–27 reads). Therefore, known porcine miRNAs with high abundance and shared between all three libraries were used for the following analysis. In addition, the small RNA sequencing data showed a significant positive correlation with qRT-PCR results (Pearson’s *r* = 0.780, *p* < 10^−6^), highlighting the reliability of the small RNA-sequencing approach (Figure S1b).

### 2.3. Universally Abundant miRNAs across the Three Muscle Types Are Associated with the Metabolic Pathways of Myogenesis and Angiogenesis

**Figure 2:**
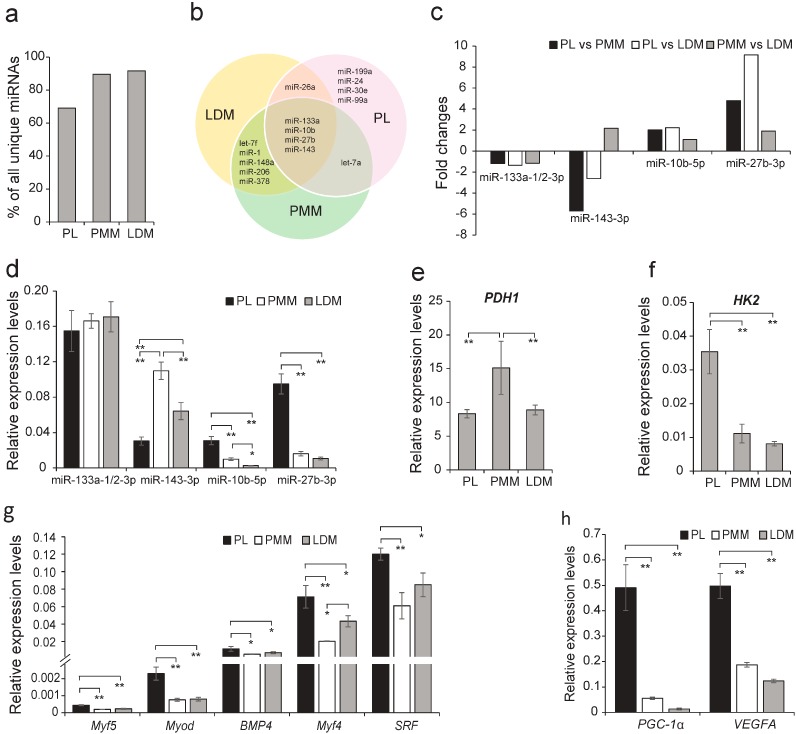
Analysis of miRNAs universally abundant across the three muscles. (**a**) The top 10 miRNA reads as a percentage of total unique miRNAs; (**b**) Distribution of the top 10 miRNAs in each muscle; (**c**) Fold change of miRNAs abundantly expressed in all three muscles. The relative expression levels, measured by qRT-PCR, of: (**d**) miRNAs shown in (**c**); (**e**) *HK2*; (**f**) *PDH1*; (**g**) myogenic regulatory factors; and (**h**) angiogenesis-related genes. Data are means ± SD. Statistical significance was calculated by one-way repeated-measures analysis of variance (*n* = 3 per individual). * *p* < 0.05, ** *p* < 0.001.

A small number of miRNAs dominated the total miRNA pool [19], thus we first analyzed the most abundantly expressed (top 10) unique miRNAs in each library. The top 10 unique miRNAs with high abundance accounted for more than 70% of the total unique miRNA reads (Figure 2a), indicating that they might play important regulatory roles in the functional maintenance of skeletal muscle (e.g., proliferation and differentiation). Notably, four miRNAs (miR-133a, miR-143, miR-27b, and miR-10b) were in the top 10 most abundant miRNAs in all three libraries (Figure 2b–d).

miR-133a, a muscle-specific miRNA involved in myogenesis, showed little difference (<1.5-fold) among the three libraries [20], indicating it may play a potential housekeeping role in the three muscle tissues [21,22]. In contrast, miR-143 was differentially expressed (>1.5-fold) among the three libraries, suggesting it might be a dominant miRNA contributing to the physiological functional differences between the fiber types. Through analysis of its target genes (Table S4), we found that miR-143 was primarily involved in metabolic pathways (e.g., mitochondrial, pyruvate metabolism, glycolysis/gluconeogenesis) [23]. In addition, the differential expression of mtDNA copy number (Figure 1b) and the metabolic pathway marker genes (*i.e.*, pyruvate metabolism: *LDHA* (Figure 1c), *PDH1* (Figure 2e); glycolysis: *HK2* (Figure 2f) [3,24,25,26,27,28]) demonstrated the different metabolic activities of the three muscle fibers.

Interestingly, compared with PMM and LDM, both miR-27b and miR-10b were upregulated (>1.5-fold) in PL. miR-27b was found to be upregulated during myogenic differentiation and directly targets *Pax3* and *MSTN* [29,30]. miR-10b was also found to be a regulator of myogenesis [25]. Taken together, we propose that there might exist certain differences in myogenesis among the three muscles. Furthermore, the myogenesis marker genes (*Myf4*, *MyoD*, *BMP4*, *Myf5*, and *SRF*) [31,32,33,34], were significantly more highly expressed in PL, which confirmed that PL had higher myogenesis capacity (Figure 2g). The activation of myogenic progenitors (e.g., satellite cells) contributed to the myogenensis of adult skeletal muscle tissues [35], thus we propose that the PL may contain more active myogenic progenitors relative to PL and LDM. Moreover, miR-10b is required for angiogenesis by indirectly inducing extracellular matrix remodeling and cell migration [36,37]. *PGC-1*α and *VEGFA* were proved to play an important role in angiogenesis [38,39,40,41,42], and in this study these two genes showed a significantly higher expression in PL (Figure 2h), suggesting that PL possesses a higher capillary load.

In summary, analysis of the four miRNAs abundantly expressed in all three muscles (miR-133a, miR-143, miR-27b, and miR-10b) not only suggests common characteristics of skeletal muscle, but also points to differences between the different fiber types with regard to metabolic pathways (e.g., mitochondrial, pyruvate metabolism, glycolysis/gluconeogenesis), myogenesis, and angiogenesis (Figure 1c and Figure S2). Further investigation is encouraged to better understand the influence of miRNAs on the phenotypes of fiber types.

### 2.4. Identification and Functional Analysis of Differentially Expressed miRNAs among Three Muscle Fibers

**Figure 3:**
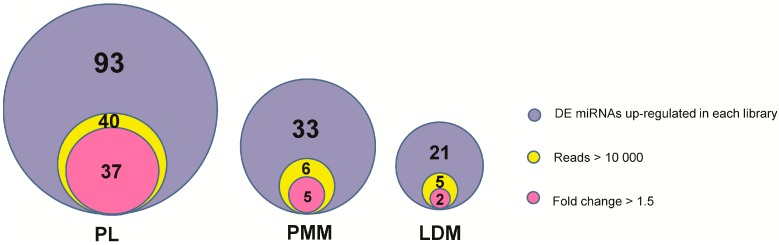
Distribution of differentially expressed miRNAs among three tissues.

To further compare the miRNA expression patterns among the three muscles, we analyzed, using IDEG6, the 292 known porcine miRNAs that were found expressed in all three muscles. Of these 292, 155 were found differentially expressed (DE) (*p* < 0.001) among the three libraries (Table S5). It is well known that miRNAs function in a dose-dependent manner [4], thus the higher abundance miRNAs (reads ≥ 10,000) were considered to be more important. Therefore, in addition to the four “top 10” miRNAs shared by all three muscles (miR-133a, miR-143, miR-27b, and miR-10b), 44 miRNAs were identified as both high abundance and upregulated in one of three tissues (>1.5-fold when compared with the other two libraries, simultaneously) and used for subsequent functional analysis (Figure 3). There were 37 DE miRNAs enriched in PL, but few miRNAs enriched in PMM or LDM. This may be explained by the high similarity of the expression patterns of PMM and LDM (Figure S3). For functional enrichment analysis, we gathered target information for the upregulated DE miRNAs from previous reports that had experimentally validated these targets in muscle tissues and/or cells (Table S6). The target genes of the remaining miRNAs, whose functions had not been previously reported, were predicted using the specific algorithms of MiRanda and TargetScan software based on our in-house dataset of the porcine skeletal muscle 3' untranslated region (UTR) and a previous report [23] (Table S7).

### 2.5. The Differentially Expressed (DE) miRNAs Involved in Myogenesis

**Figure 4:**
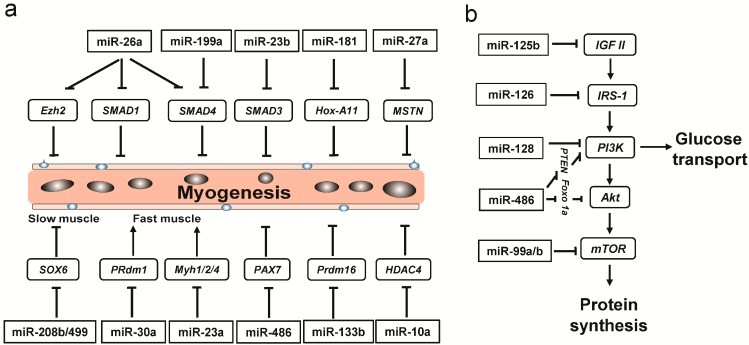
Schematic of the regulatory mechanism for myogenesis by PL-enriched miRNAs via targeting (**a**) myogenic regulatory factors and (**b**) insulin-like growth factor (IGF) pathways. The blue and black circles in (**a**) indicated satellite cells and cell nucleus in the myofiber (light brown tube), respectively.

We found 18 DE miRNAs implicated in myogenesis based on functional enrichment analysis of their target genes. Of these, 12 miRNAs were specifically enriched in PL (Figure 4a). Interestingly, of the 12 PL-enriched miRNAs, four miRNAs (miR-499, miR-208b, miR-30a and miR-23a) could increase the proportion of oxidative red fiber type (*i.e.*, slow-type) by targeting *Sox6* (repressor of slow-twitch genes) [43], *Myh1/2/4* (fast-twitch genes), or *Prdm1* (promote fast muscle formation) [44,45]. In addition, these 12 PL-enriched miRNAs were predicted to target the TGF-beta signaling pathway (Table S7), which plays an important role in myogenesis [46]. In agreement with previous reports, three PMM-enriched miRNAs (miR-378, miR-148a, and miR-101) and two LDM-enriched miRNAs (miR-1 and miR-885) were also identified to play a vital role in myogenesis (Table S6).

Numerous studies indicate that the insulin-like growth factor (IGF) pathways act as positive regulators of myogenesis [32,47,48]. We found six PL-enriched miRNAs (miR-125b, miR-126, miR-128, miR-486, and miR-99a/b) involved in the insulin signaling pathway (Figure 4b). miR-486 promoted the insulin signaling pathway [49], while the others, especially miR-128 (>14-fold), miR-99a (>25-fold), and miR-99b (>15-fold) repressed the insulin signaling pathway. To further study downstream effects on this pathway, we measured the relative expression levels of four insulin signaling pathway marker genes (*IGF1*, *PI3K*, *Akt1*, and *mTOR*) (Figure S4). *IGF1* and *PI3K* exhibited higher expression levels in PL, which indicated that PL muscle is better able to stimulate glucose transport [5,50]. In contrast, the expression levels of *Akt1* and *mTOR*, which promote skeletal muscle hypertrophy, especially in fast muscle [51,52], were significantly higher in PMM. Combining the miRNA and mRNA expression data, these conflicting results indicate that regulation of insulin signaling is a complicated process in these three tissues. Nonetheless, we identified numerous DE miRNAs that were involved in myogenesis, and remarkably, most of them were especially enriched in PL and directly targeted slow muscle repressors or fast muscle genes, indicating their vital roles in the development of oxidative red muscle. The different expression levels of myogenesis marker genes confirmed that PL had higher muscle hypertrophy and differentiation capacity.

### 2.6. Energy Metabolism-Related miRNAs Enriched in PL

**Figure 5:**
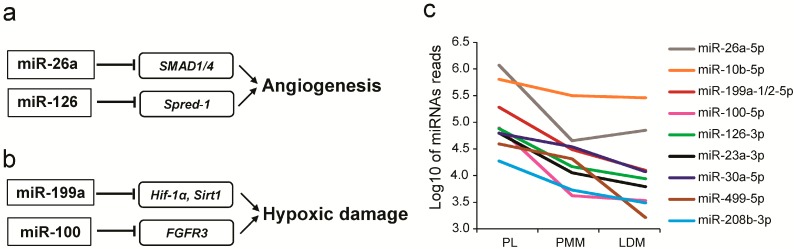
Energy metabolism-related miRNAs enriched in PL. miRNAs involved in (**a**) angiogenesis and (**b**) reducing hypoxic damage; (**c**) Abundance of energy metabolism-related miRNAs.

Interestingly, we found that two angiogenesis-related miRNAs (miR-26a and miR-126), shown to be involved in angiogenesis by targeting *SMAD1/4* [53] and *Spred-1* [54], respectively, were highly expressed in PL (Figure 5a), suggesting that PL contains abundant capillaries. Additionally, we also found that two miRNAs (miR-100 and miR-199a, >10-fold), known to be involved in reducing hypoxic damage by targeting *Hif-1α*/*Sirt1* and *FGFR3* [55], respectively (Figure 5b), were significantly enriched in PL, suggesting that PL might have a relatively higher oxygen content than the other muscles. Combined with our above results, nine miRNAs mainly associated with angiogenesis (miR-10b, miR-26a, and miR-126), reducing hypoxic damage (miR-100 and miR-199a) and slow muscle formation (miR-499, miR-208b, miR-30a, and miR-23a) were found highly expressed in PL (Figure 5c). Among them, two miRNAs implicated in linking muscle fiber type to energy metabolism [11,46], were highly expressed in PL: miR-208b, encoded within the intron of *Myh7* gene (Figure S5a), and miR-499, encoded within the intron of *Myh7b* (*i.e.*, another encoded the slow-tonic MHC gene [56]) (Figure S5b). Collectively, these miRNAs play a critical role in energy metabolism for red fibers, through enhanced capillary load. This could result in transport of more nutrients (e.g., glucose) and oxygen, and, coupled with higher levels of mitochondrial content, could result in improved glucose use for mitochondrial oxidative metabolism.

It is worth noting that most of our above hypothesis/conclusions are based on the previous reports from multiple model organisms, and there are still some conflicting results between our hypothesis and previous reports from other organisms. For example, the *Prdm1* gene, which is regarded as an activator of the fast muscle program in our current study, was proved to promote the slow muscle formation in zebrafish [57]. Notably, a recent study in mouse indicated that there was no conservation of function for the evolutionarily conserved *Prdm1* in the control of the slow twitch myogenic program between teleosts and mammals [58]. It is therefore reasonable to assume that these conflicting results may be due to the species-specific molecular regulation network. Further studies focusing on species-specific regulation of miRNAs are needed to elucidate the complicated epigenetic mechanism underlying the formation- and function-variations among distinct muscle types.

## 3. Experimental Section

### 3.1. Animal Ethics Statement

All research involving animals was conducted according to the Regulations for the Administration of Affairs Concerning Experimental Animals (Ministry of Science and Technology, China, revised in June 2004) and approved by the Institutional Animal Care and Use Committee in the College of Animal Science and Technology, Sichuan Agricultural University, Sichuan, China under permit No. DKY-B20110807. Animals were allowed access to food and water *ad libitum* under normal conditions, and were humanely sacrificed as necessary, to ameliorate suffering.

### 3.2. Animals and Tissue Collection

Given the plasticity and maturation processes of porcine myofiber [59], the 210 days old female Landrace pigs, that were in the young adult stage of their lifespan [60,61] with stable myofiber composition in skeletal muscles, were selected as research objects to investigate the miRNA transcriptome variations underlying the physiological and biochemical differences of porcine distinct skeletal muscle types. Fifteen muscles were obtained from three female Landrace pigs (210 days old), and immediately frozen in liquid nitrogen, then stored at −80 °C until RNA extraction. These 15 porcine skeletal muscles were collected in different anatomic locations: *longissimus dorsi* muscle (LDM), *psoas major* muscle (PMM), *gluteus medius* (GMED), *latissimus dorsi* (LD), *gastrocnemius* (GC), *peroneal longus* (PL), *soleus* (S), *semitendinosus* (ST), *masseter* (M), *triceps brachii* (TB), *obliquus externus abdominis* (OEA), *trapezius* (T), *semimembranosus* (SM), *biceps femoris* (BF), *semispinalis capitis* (SC).

### 3.3. Quantitative Real-Time PCR: mRNA, miRNA Expression, and mtDNA Copy Number

Total RNA was isolated from the muscle tissues using TRIzol reagent (Takara, Dalian, China) according to the manufacturer’s protocol. For mRNA, cDNA was synthesized using the PrimeScript RT Master Mix kit (Takara) following the manufacturer’s recommendation. qRT-PCR was performed using the SYBR Premix Ex Taq kit (Takara) on a CFX96 Real-Time PCR detection system (Bio-Rad Laboratories, Richmond, CA, USA). Porcine *PPLA*, *RPL4* and *YWHAZ* were simultaneously used as mRNA endogenous control genes [62]. The mRNA primers are shown in Table S8. For miRNA, the expression levels were validated using the SYBR PrimeScript miRNA RT-PCR Kit (Takara) on the CFX96 Real-Time PCR Detection System. Three miRNA endogenous control genes (*U6* snRNA, *18S* rRNA, and *5S* rRNA) were used in this assay [63]. The forward primer of miRNAs was identical in sequence and length to the miRNA itself (*i.e.*, the most abundant isomiR) based on our sequencing results. The 2^−ΔΔ*C*t^ method was used to calculate the relative expression levels of mRNAs and miRNAs.

The ratio of MHC isoforms was expressed as [64]:

[MHC isoform/(*Myh1* + *Myh2* + *Myh4* + *Myh7*) × 100%] (1)

Total DNA was isolated from muscle tissues using TIANamp Genomic DNA Kit (Tiangen, Beijing, China). The number of mtDNA copies/cell was quantified using qRT-PCR as previously described [65]. We selected three mitochondrial DNA-specific genes (*ATP6*, *COX1*, and *ND1*) and a single-copy nuclear DNA gene (glucagon gene, *GCG*) [66] to calculate the number of mtDNA copies per cell using the following formula:

[(No. of copies of the mtDNA gene)/(No. of copies of *GCG*)] (2)

### 3.4. Validation of the Specificity of Myosin Heavy Chain Gene Primers

For four MHC genes (*Myh1*, *Myh2*, *Myh4*, *Myh7*), the presence of PCR products was confirmed by 2% agarose gel electrophoresis. Subsequently, the PCR products were cloned into the pMD19-T Vector (Takara), and then randomly selected clones (*n* = 3) were sequenced using Sanger sequencing approach to validate the specificity of the PCR products (Huada Company, Beijing, China).

### 3.5. Small RNA Library Construction and Sequencing

Total RNA was extracted from PL using the mirVana™ miRNA isolation kit (Ambion, Austin, TX, USA) following the manufacturer’s procedure. The quantity and purity of total RNA were monitored by NanoDrop ND-1000 spectrophotometer (Nano-Drop Technologies, Wilmington, DE, USA) at 260/280 nm (ratio > 2.0). The integrity of total RNA was monitored by the Bioanalyzer 2100 and RNA 6000 Nano LabChip Kit (Agilent Technologies, Palo Alto, CA, USA) with RIN number > 6.0. Equal quantities (5 µg) of small RNA isolated from three female Landrace pigs were pooled. Briefly, approximately 15 µg of small RNA was used for library construction and sequencing. Small RNA fragments (between 10 and 40 nt) were isolated by polyacrylamide gel electrophoresis (PAGE) and ligated with proprietary adaptors (Illumina, San Diego, CA, USA). The small RNA fractions were then converted to cDNA by RT-PCR and the cDNA was sequenced on the Genome Analyzer GA-II (Illumina) following the recommended manufacturer’s protocol. The small RNA sequence data have been uploaded to NCBI’s Gene Expression Omnibus (GEO) (Accession Number GSE64523).

### 3.6. In Silico Analysis of Small RNA-Sequencing Data

The small RNA-sequencing data of LDM and PMM were obtained from the same pigs in our previous study [9]. The raw reads of all these three tissues (PL, PMM, and LDM) were then subjected to a series of additional strict filters (*i.e.*, the following reads were removed: 3' adapter not found; length less than 16 bases or more than 29 bases; junk reads). Then the high-quality reads were mapped to the pig genome (Sscrofa10.2) using NCBI Local BLAST following three steps in order: (1) map the high-quality reads to the precursor miRNAs of pig and 24 other mammals in miRBase 19.0; (2) map the mapped high-quality reads to pig genome (Sscrofa10.2) to obtain their genomic locations and annotations using NCBI Local BLAST; (3) cluster the unmapped sequences in step 1 that mapped to the pig genome as putative novel miRNAs, and predict their hairpin RNA structures from the adjacent 60 nt sequences in either direction from the pig genome using UNAFold [67].

### 3.7. Differentially Expressed (DE) miRNAs

The expression of miRNAs in the three samples was normalized by total mappable reads, and the program IDEG6 was employed to detected DE miRNAs among the three libraries (http://telethon.bio.unipd.it/bioinfo/IDEG6_form/). A unique miRNA is considered to be differentially expressed when it simultaneously obtained *p* < 0.001 under three statistical tests (Audic-Claverie test, Fisher exact test and Chi-squared 2 × 2 test with Bonferroni correction by pairwise comparison).

### 3.8. Functional Analysis

Three prediction programs (PicTar [68], TargetScan human 6.2 [69], and MicroCosm Targets Version 5.0 [70]) were used to predict target genes of miRNAs, and the intersection of results from the three programs comprised the final predicted targets. The predictions were made based on the interactions of human mRNA-miRNA, as porcine miRNAs were not available in the current version of the above-mentioned algorithms. The gene ontology (GO), biological process (BP), molecular function (MF), cellular component (CC) terms, and KEGG pathways enriched for predicted target genes were determined using the DAVID bioinformatics resources [56].

## 4. Conclusions

In this study, we determined the muscle fiber composition of 15 types of porcine muscle tissues derived from distinct anatomical locations, and classified them into red, intermediate, and white muscle types. Then, the *peroneal longus* muscle (PL), *psoas major* muscle (PMM), and *longissimus dorsi* muscle (LDM) were selected as the typical tissues for red, intermediate, and white muscle types, and subjected to miRNA transcriptome investigation. As a result, muscle type-specific enriched miRNAs were identified and implicated in promoting the specific formation of distinct muscle fibers. DE and functional enrichment analysis showed that the DE miRNAs among distinct muscle types were mainly related to low-oxidative myofiber formation, angiogenesis, energy metabolism, and reduced hypoxic damage, which reflected the intrinsic characteristics of the physiological and metabolic roles of different muscle types. In addition, the expression pattern of a set of miRNAs (miR-10b, miR-26a, miR-126, miR-199a, miRNA-208b, and miRNA-499) linked the capacity of myogenesis and energy metabolism levels with distinct fiber types. Our study performed here will aid the further understanding of miRNAs with their biological functions in different muscle fiber types.

## Supplementary Materials

Supplementary materials can be found at http://www.mdpi.com/1422-0067/16/05/9635/s1.
