# Supplementary material for: The miRNA Transcriptome Directly Reflects the Physiological and Biochemical Differences between Red, White, and Intermediate Muscle Fiber Types
Source: Int J Mol Sci. 2015 Apr 29;16(5):9635–53. doi: 10.3390/ijms16059635 (PMC4463610; doi:10.3390/ijms16059635)
Supplement: Supplementary file 1 [file ijms-16-09635-s001.zip › ijms-75212-Supplementary Information-Figures.pdf]

Supplementary Information

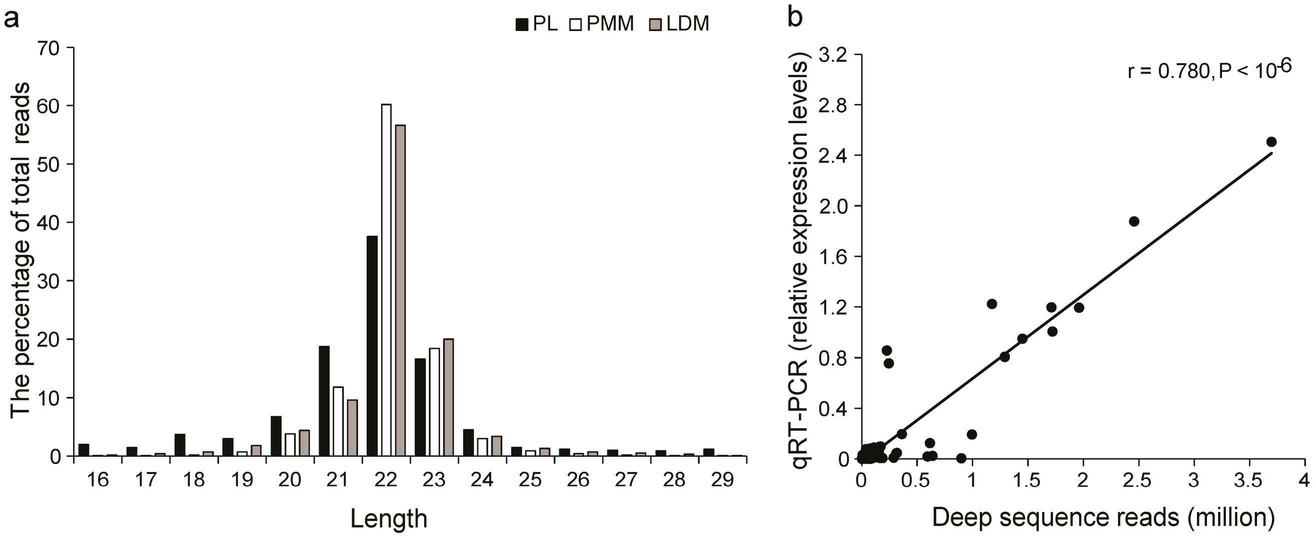

Figure S1. (**a**) Length distribution of the mappable reads; (**b**) q-PCR validation for a randomly selected set of 20 miRNAs. Pearson's correlation was used to determine the relationship between the q-PCR and small RNA-seq results for miRNA expression levels.

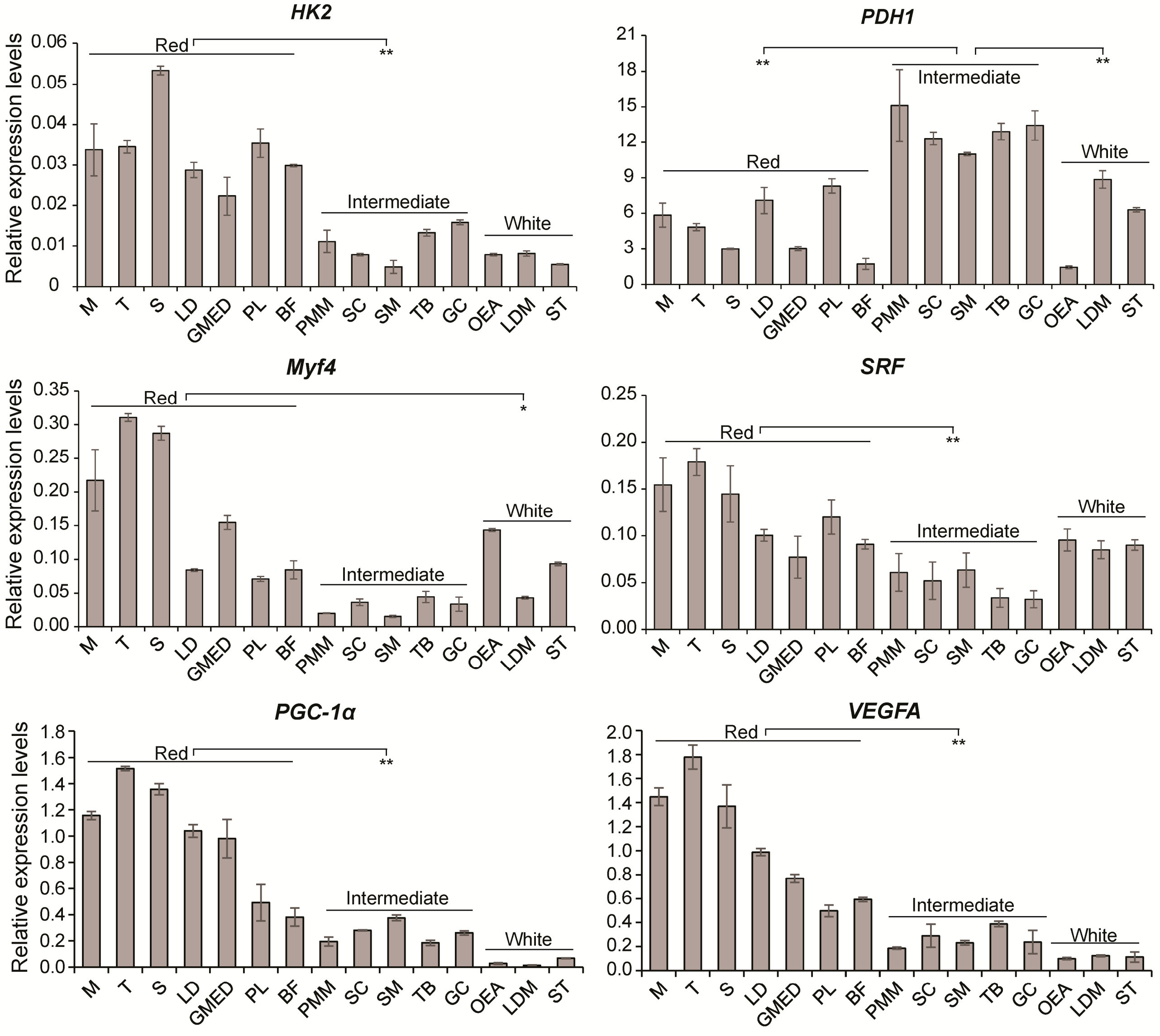

Figure S2. The expression profiling of marker genes for metabolic pathways (*HK2* and *PHD1*), myogenesis (*Myf4* and *SRF*) and angiogenesis (*PGC-1α* and *VEGFA*) across distinct muscle tissues. Data are means ± SD. Statistical significance was calculated by one-way repeated-measures analysis of variance. * $p < 0.05$, ** $p < 0.001$.

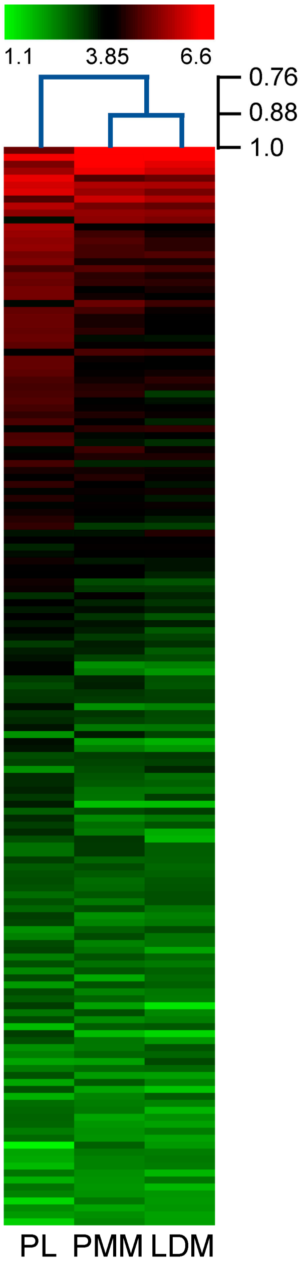

Figure S3. Hierarchical clustering analysis for three muscle types based on the expression of 365 Known porcine miRNAs.

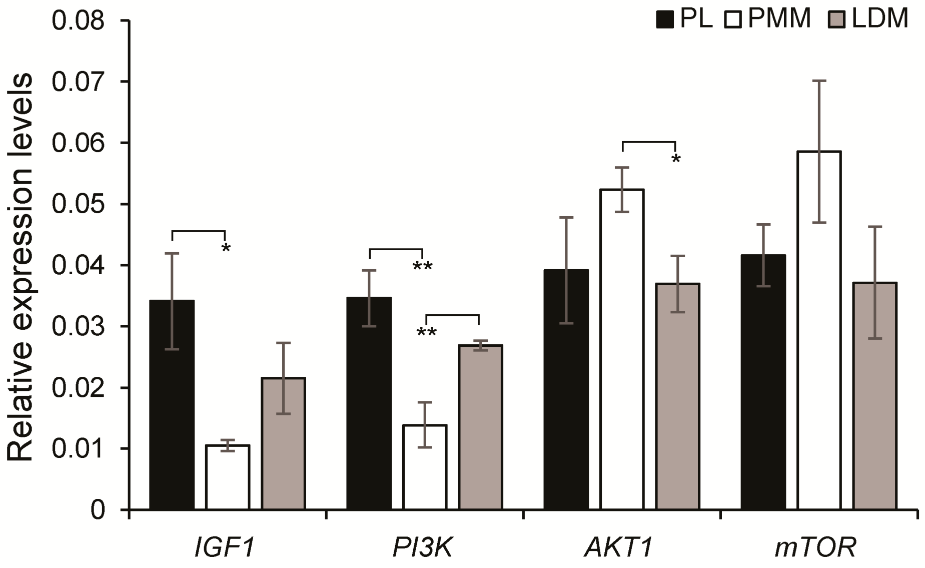

Figure S4. The relative expression levels of four insulin signaling pathway marker genes across three distinct muscle types. Data are means ± SD. Statistical significance was calculated by one-way repeated-measures analysis of variance. * $p < 0.05$, ** $p < 0.001$.

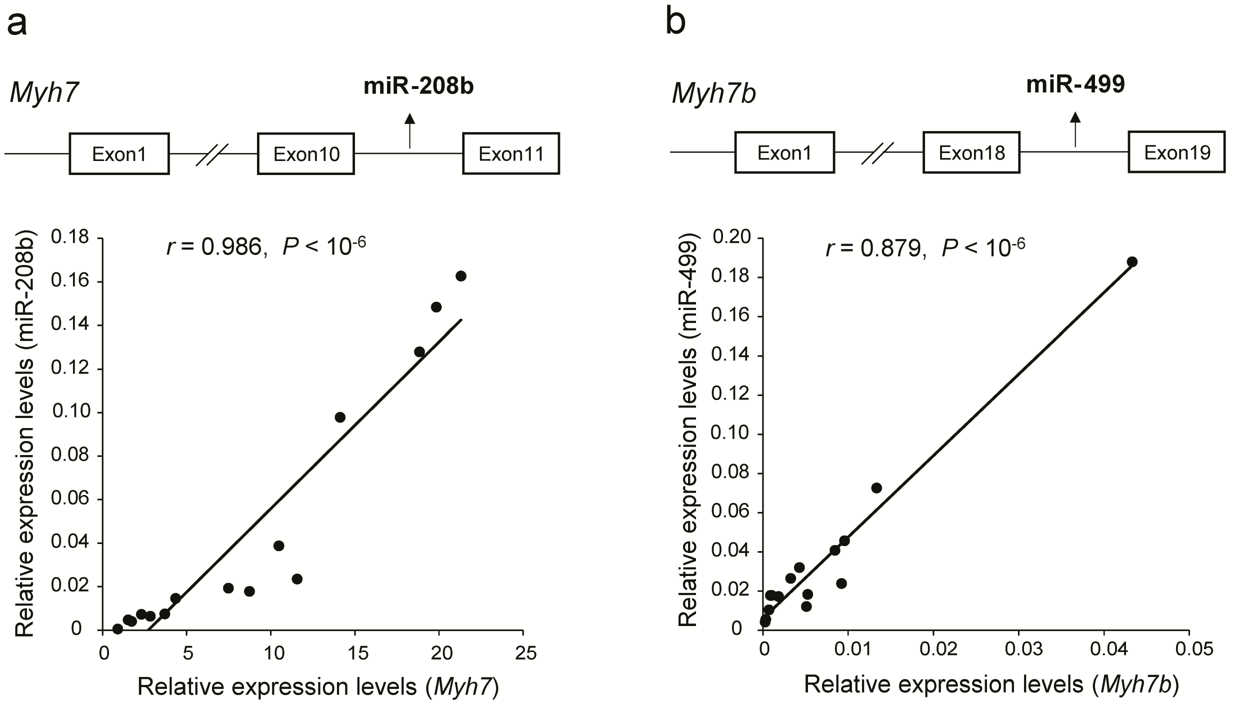

Figure S5. (**a**) The genomic relationship and expression correlation between miR-208b and its host gene, *Myh7*; (**b**) The genomic relationship and expression correlation between miR-499 and its host gene, *Myh7b*.
