# Supplementary material for: The miRNA Transcriptome Directly Reflects the Physiological and Biochemical Differences between Red, White, and Intermediate Muscle Fiber Types
Source: Int J Mol Sci. 2015 Apr 29;16(5):9635–53. doi: 10.3390/ijms16059635 (PMC4463610; doi:10.3390/ijms16059635)
Supplement: Supplementary file 1 [file ijms-16-09635-s001.zip › ijms-75212-Supplementary Information-Tables.zip!/ijms-75212-Supplementary Information-Tables/Table S6.pdf]

Supplementary Information

Table S6. Target information of DE miRNAs gathered from previous reports and bioinformatic prediction.

No.	miRNA Name	PL up Fold Change		Target Genes or Pathways
		GC *vs.* LDM	GC *vs.* PMM	
1	ssc-miR-26a-5p	16.67	26.05	Ezh2 [71], (Smad1,Smad4) [72]
2	ssc-let-7a-1/2-5p	5.74	3.81	Predicted
3	ssc-miR-99a-5p	37.65	25.46	mTOR [73]
4	ssc-miR-199a-1/2-5p	15.25	6.23	(Hif-1α, Sirt1) [74]
5	ssc-miR-30e-5p	7.91	4.81	Ubc9 [75], UCP2 [76]
6	ssc-miR-24-1/2-3p	7.58	3.89	promotes skeletal muscle differentiation [77]
7	ssc-miR-191-5p	7.88	7.91	Predicted
8	ssc-miR-151-5p	6.49	15.91	Predicted
9	ssc-miR-133b-3p	2.09	2.50	Prdm16 [78]
10	ssc-miR-27a-3p	15.75	7.16	Pax3 [29], myostatin [79–81]
11	ssc-let-7g-5p	5.35	3.94	Predicted
12	ssc-miR-100-5p	23.19	18.71	FGFR3 [82]
13	ssc-let-7i-5p	10.11	5.10	Predicted
14	ssc-miR-126-3p	8.70	5.15	IRS-1 [83], Spred-1 [54], VCAM-1 [84]
15	ssc-miR-30c-1/2-5p	3.19	2.80	Predicted
16	ssc-miR-10a-5p	10.39	3.19	HDAC4 [85]
17	ssc-miR-125a-5p	10.98	9.46	Predicted
18	ssc-miR-23a-3p	10.10	5.57	(MAFbx/atrogin-1, MuRF1) [86], Myh1/2/4 [44]
19	ssc-miR-30a-5p	5.23	1.79	PRDM1 [45], DLL4 [87]
20	ssc-miR-125b-1-5p	6.49	5.86	IGF-II [88]
21	ssc-miR-30b-5p	4.03	5.20	DLL4 [89]
22	ssc-miR-374a-5p	12.03	7.72	Predicted
23	ssc-miR-199a-1/2-3p	21.57	12.26	(Hif-1α, Sirt1) [74]
24	ssc-miR-126-5p	15.65	4.71	IRS-1 [83], Spred-1 [54], VCAM-1 [84]
25	ssc-miR-181a-1/2-5p	1.80	3.34	Hox-A11 [90]
26	ssc-miR-499-5p	24.08	1.91	(Thrap1, Sox6 and Purb) [43]
27	ssc-miR-486-2-5p	6.25	8.65	(PTEN , Foxo1a) [91], Pax7 [92]
28	ssc-miR-30d-5p	2.60	2.04	RUNX2 [93]
29	ssc-miR-23b-3p	4.14	3.43	Smad3 [94]
30	ssc-miR-128-2-3p	14.34	14.67	IRS1/Akt [95]
31	ssc-miR-99b-5p	16.74	15.65	TNF-α [96], mTOR [97]
32	ssc-let-7c-5p	6.30	2.90	IGF1R [98]
33	ssc-let-7d-5p	6.85	4.43	Predicted
34	ssc-miR-208b-3p	6.11	3.51	sox6 [43]
35	ssc-miR-1271-5p	15.57	13.58	Predicted
36	ssc-miR-98-5p	3.65	3.87	cyclin D2 [99]
37	ssc-let-7e-5p	7.40	7.06	Predicted

Table S6. *Cont.*

No.	miRNA Name	PMM up Fold Change		Target Genes or Pathways
		**PMM *vs.* LDM**	**PMM *vs.* PL**	
1	ssc-miR-378-1/2-3p	1.73	15.83	MyoR [100], (BMP2, MAPK1) [101], PGC-1α [102]
2	ssc-miR-148a-3p	1.98	14.61	ROCK1 [103]
3	ssc-miR-101-1/2-3p	2.56	16.64	EZH2 [104]
4	ssc-miR-381-3p	2.77	6.54	Metabolic pathways (Table S7.1) [23]
5	ssc-miR-30e-3p	1.80	2.00	Ubc9 [75], UCP2 [105]
**No.**	**miRNA Name**	**LDM up Fold Change**		**Target Genes or Pathways**
		**LDM *vs.* PMM**	**LDM *vs.* PMM**	
1	ssc-miR-1-3p	1.50	51.48	(HDAC4, SRF) [21], MSTN [106] *etc.*
2	ssc-miR-885-5p	4.90	5.34	related to skeletal development [10]
